# Inflammatory phenotypes and clinical outcomes amongst patients with presumed and confirmed *Pneumocystis jirovecii* pneumonia without underlying human immunodeficiency virus infection

**DOI:** 10.1093/femspd/ftaf005

**Published:** 2025-04-24

**Authors:** Matthew Chung Yi Koh, Lionel Hon-Wai Lum, Paul Anantharajah Tambyah, Jinghao Nicholas Ngiam

**Affiliations:** Department of Medicine, Division of Infectious Diseases, National University Hospital, National University Health System, Singapore 119228, Singapore; Department of Medicine, Division of Infectious Diseases, National University Hospital, National University Health System, Singapore 119228, Singapore; Department of Medicine, Division of Infectious Diseases, National University Hospital, National University Health System, Singapore 119228, Singapore; Department of Medicine, Division of Infectious Diseases, National University Hospital, National University Health System, Singapore 119228, Singapore

**Keywords:** *Pneumocystis pneumonia*, inflammatory phenotype, mortality, non-HIV

## Abstract

*Pneumocystis jirovecii* pneumonia (PJP) has significant mortality, especially in immunocompromised hosts without underlying human immunodeficiency virus infection (HIV). Inflammatory phenotypes may influence clinical outcomes. This study examines the relationship between inflammation, as measured by C-reactive protein (CRP), and adverse outcomes in HIV-negative patients with PJP. A retrospective analysis was conducted on 62 HIV-negative patients with presumed or confirmed PJP from 2006 to 2023. CRP measured within 48 hours of admission were used to classify patients into hypo-inflammatory (CRP<30 mg/l), normo-inflammatory (30–135 mg/l), and hyper-inflammatory (>135 mg/l) groups. Composite adverse outcomes (defined as all-cause in-hospital mortality or mechanical ventilation) were compared across groups using univariate and multivariable analyses. The inflammatory groups differed in CRP but not significantly in terms of white cell count, ferritin, or lactate dehydrogenase. Corticosteroid use was similar across groups. Adverse outcomes occurred in 37.5% of the hypo-inflammatory group, 20.0% of the normo-inflammatory group, and 68.8% of the hyper-inflammatory group (*P* = 0.005). Hyper-inflammation independently predicted adverse outcomes (adjusted OR 6.98, 95% CI 1.81–26.92, *P* = 0.005). This study raises the possibility of a U-shaped relationship between inflammatory phenotypes and outcomes in PJP, with hypo- and hyper-inflammatory phenotypes associated with worse outcomes.

## Teaser

Inflammatory phenotypes in *Pneumocystis jirovecii* pneumonia independently predict adverse outcomes in HIV-negative immunocompromised hosts, which may have implications on adjunctive corticosteroid therapy.

## Introduction

*Pneumocystis jirovecii* pneumonia (PJP) can be severe and fulminant in non-human-immunodeficiency virus (HIV)-infected hosts (Bollée et al. 2007, Liu et al. 2017, Asai et al. 2012). A broad range of immunosuppressive conditions, ranging from haematological malignancies, solid organ cancers on chemotherapy, corticosteroid use for inflammatory conditions and other novel immunomodulatory therapies may place an individual at risk for PJP (Maini et al. 2013, Nasr et al. 2022). In fact, due to the increase in use of these immunosuppressive therapies, PJP has been on the rise (Maini et al. 2013). Importantly, the mortality for this condition, in individuals without HIV is high, and can be in excess of 30% (Liu et al. 2017, Wang et al. 2022). We had previously reported that older age, male sex and elevated lactate dehydrogenase levels at presentation correlated with adverse outcomes in non-HIV patients hospitalized with PJP (Koh et al. 2024).

In terms of therapeutics, the guidance on the management of PJP in non-HIV is largely extrapolated from the experience treating PJP in persons living with HIV (Walzer et al. 2008). For example, the use of adjunctive corticosteroids is commonly recommended in patients with PJP and a significant degree of hypoxia. In non-HIV infected hosts, it is unclear if adjunctive corticosteroids confer similar survival benefit (Fujikura et al. 2017). In other meta-analyses, subgroups of patients with significant degree of respiratory failure did appear to benefit from adjunctive corticosteroid administration (Inoue and Fushimi, 2019, Ding et al. 2020). However, if not required, adding additional corticosteroids to an already profoundly immunosuppressed patient may place them at an unnecessary elevated risk for other opportunistic infections.

We hypothesize that the group of non-HIV infected hosts who develop PJP are heterogenous. There may be subgroups that could be stratified by degree of inflammatory response to PJP, and these subgroups may have vastly different clinical outcomes. We thus sought to define inflammatory phenotypes in PJP amongst non-HIV infected hosts, describing differences in their clinical presentations and outcomes.

## Methods

Thus, we examined consecutive patients diagnosed with PJP, admitted to a single tertiary centre from 2006 to 2023, who tested negative for HIV. All patients also had a C-reactive protein (CRP) test done within 48 hours of admission, to help stratify them based on their inflammatory phenotype. PJP was defined by a compatible clinical syndrome, with fever, cough or sputum, or shortness of breath, in an immunocompromised host as defined by the revised EORTC/MSGERC disease definitions, with subsequent confirmation either by i) direct microscopy with identification of the organism with silver staining from sputum or bronchoalveolar lavage (proven PJP), ii) positive PJP polymerase chain reaction (PCR) testing from sputum or bronchoalveolar lavage (probable PJP) (Lagrou et al. 2021). Additionally, we included another group of patients with in the appropriate host and clinical syndrome as above, where a presumptive diagnosis of PJP was made based on compatible radiographic features (bilateral interstitial infiltrates on chest imaging) and a clinical response to PJP directed therapy.

The cohort was stratified based into three groups by CRP centiles, namely the hypo-inflammatory (up to 25th centile), normo-inflammatory (25th to 75th centile) and hyper-inflammatory (above 75th centile) groups. We had used a similar approach to define inflammatory phenotypes in patients with COVID-19 previously (Ngiam et al. 2023). We compared baseline demographic, clinical and laboratory data, as well as clinical outcomes. Composite adverse outcomes were defined as the need for mechanical ventilation or all-cause in-hospital mortality. Categorical variables were compared using Chi-squared tests, while continuous variables were compared by one-way analysis of variance. A multivariable model was subsequently constructed to identify independent predictors of adverse outcomes. All statistical analyses for this study were performed on SPSS version 20.0 (SPSS, Inc., Chicago, Illinois). A *P*-value <0.05 was considered significant. This study had been approved by the National Healthcare Group Domain Specific Review Board (DSRB 2023/00846), and was conducted in accordance with the principles laid out by the Declaration of Helsinki.

## Result

We examined a total of 62 consecutive patients with PJP. By CRP, these were stratified into the hypo-inflammatory group (CRP below the 25th centile, <30 mg/l; *n* = 16), normo-inflammatory group (CRP from 25th to 75th centile, 30–135 mg/l; *n* = 30), and the hyper-inflammatory group (CRP above the 75th centile, >135 mg/l, *n* = 16). These groups did not differ in terms of age, sex, or medical co-morbidities such as hypertension, hyperlipidaemia, or diabetes mellitus. Across the three groups, the causes of immunosuppression also did not differ significantly between the groups. Haematological disorders and solid organ cancers were amongst the commonest causes of immunosuppression in our population. Steroid use prior to PJP also did not differ significantly, and a majority of patients did not receive PJP prophylaxis (Table 1).

**Table 1.** Clinical characteristics of patients with *Pneumocystis pneumonia* without underlying HIV, stratified by inflammatory phenotypes.

Parameter	Hypo-inflammatory (CRP<30 mg/l) (*n* = 16)	Normo-inflammatory (CRP 30-135 mg/l) (*n* = 30)	Hyper-inflammatory (>135 mg/l) (*n* = 16)	*P*-value
**Age (years)**	51.0 (±17.1)	57.7 (±16.5)	63.0 (±18.2)	0.148
**Sex (male)**	4 (25.0%)	14 (46.7%)	11 (68.8%)	0.046
**Diabetes mellitus**	2 (12.5%)	9 (30.0%)	2 (12.5%)	0.239
**Hypertension**	5 (31.3%)	9 (30.0%)	5 (31.3%)	0.994
**Hyperlipidaemia**	2 (12.5%)	6 (20.0%)	5 (31.3%)	0.421
**Ischaemic heart disease**	0 (0.0%)	1 (3.3%)	1 (6.3%)	0.606
**Comorbidity**				0.633
**Haematological malignancy**	4 (25.0%)	9 (30.0%)	3 (18.8%)	
** Bone marrow transplant**	2 (12.5%)	9 (30.0%)	3 (18.8%)	
**Solid organ transplant**	3 (18.8%)	5 (16.7%)	1 (6.3%)	
**Solid organ cancer**	3 (18.8%)	5 (16.7%)	6 (37.5%)	
**Autoimmune/inflammatory**	3 (18.8%)	8 (26.7%)	2 (12.5%)	
**Others**	1 (6.3%)	2 (6.7%)	3 (18.8%)	
**Steroid use**	12 (75.0%)	22 (73.3%)	11 (68.8%)	0.917
**Equivalent steroid use of prednisolone 20 mg for > 4 weeks**	8 (50.0%)	17 (56.7%)	7 (43.8%)	0.698
**Other immunosuppressants**	16 (100.0%)	28 (93.3%)	11 (68.8%)	0.011
Did not receive PJP*prophylaxis	15 (93.8%)	25 (83.3%)	15 (93.8%)	0.471
**Respiratory rate (per minute)**	21.9 (±4.6)	24.7 (±5.8)	22.3 (±6.0)	0.175
**Oxygen saturations (%)**	93.9 (±5.3)	94.1 (±5.4)	89.8 (±7.1)	0.055
**Total white cell count (x10^9^/l)**	6.2 (±4.6)	9.0 (±7.3)	10.7 (±6.6)	0.158
**Absolute neutrophil count (x10^9^/l)**	3.9 (±2.5)	7.1 (±7.3)	8.7 (±6.5)	0.088
**Absolute lymphocyte count (10^9^/l)**	1.2 (±1.7)	0.8 (±0.8)	1.1 (±1.3)	0.600
**Serum urea (mmol/l)**	6.5 (±5.9)	8.7 (±7.7)	8.5 (±6.5)	0.579
**Serum creatinine (µmol/l)**	91.9 (±66.9)	118.2 (±108.3)	87.5 (±60.1)	0.447
**Initial lactate dehydrogenase (U/l)**	888.1 (±470.4)	1058.8 (±983.3)	974.1 (±589.7)	0.789
**Initial C-reactive protein (mg/l)**	17.0 (±9.0)	78.9 (±29.4)	196.4 (±48.2)	<0.001
**Serum ferritin (mcg/l)**	1244 (±1946)	2442.4 (±2478)	1320 (±758)	0.676
**Serum albumin (g/l)**	34.0 (±6.7)	33.2 (±6.8)	30.7 (±3.7)	0.304
**Serum bilirubin (µmol/l)**	15.6 (±19.5)	12.9 (±11.7)	13.5 (±12.2)	0.821
**Serum aspartate transaminase (U/l)**	65.6 (±54.2)	46.0 (±32.5)	40.9 (±25.2)	0.160
**Serum alanine transaminase (U/l)**	66.9 (±68.2)	36.0 (±27.1)	31.2 (±23.0)	0.032
**Serum alkaline phosphatase (U/l)**	139.2 (±118.5)	165.7 (±268.6)	148.3 (±110.9)	0.912
**Microscopically confirmed diagnosis, or via polymerase chain reaction**	9 (56.3%)	18 (60.0%)	9 (56.3%)	0.956
** Proven PJP** *	3 (18.8%)	11 (36.7%)	5 (31.3%)	
** Probable PJP** *	6 (37.5%)	7 (23.3%)	4 (25.0%)	
**Treatment of PJP**				0.281
** No treatment**	0 (0.0%)	1 (3.3%)	0 (0.0%)	
** Trimethoprim-sulfamethoxazole**	14 (87.5%)	25 (83.3%)	16 (100.0%)	
** Clindamycin and primaquine**	2 (12.5%)	1 (3.3%)	0 (0.0%)	
** Nebulized pentamidine**	0 (0.0%)	0 (0.0%)	0 (0.0%)	
** Intravenous pentamidine**	0 (0.0%)	3 (10.0%)	0 (0.0%)	
** Atovaquone**	0 (0.0%)	0 (0.0%)	0 (0.0%)	
** Dapsone**	0 (0.0%)	0 (0.0%)	0 (0.0%)	
**Adjunctive steroid use**	13 (81.3%)	22 (73.3%)	15 (93.8%)	0.248
**Required intensive care**	9 (56.3%)	12 (40.0%)	8 (50.0%)	0.550
**Required mechanical ventilation**	6 (37.5%)	4 (13.3%)	6 (37.5%)	0.094
**Length of intensive care unit stay (days)**	17.3 (±15.2)	11.5 (±10.2)	17.3 (±12.8)	0.487
**Mortality**	1 (6.3%)	4 (13.3%)	10 (62.5%)	<0.001
**Composite adverse outcomes (death or mechanical ventilation)**	6 (37.5%)	6 (20.0%)	11 (68.8%)	0.005

* PJP: *Pneumocystis jirovecii* pneumonia.

In terms of initial laboratory findings, despite markedly different levels of CRP that stratified the groups, other markers of inflammation such as the total white cell count, absolute neutrophil count, lactate dehydrogenase levels and serum ferritin levels did not differ significantly between the groups. A majority of patients were treated with trimethoprim-sulfamethoxazole (TMP-SMX), and received adjunctive corticosteroids as part of PJP therapy, which was similar across the three groups. In our cohort, a total of 15 patients required a switch in drug therapy. Two patients were switched to TMP-SMX (from clindamycin and primaquine), three patients were switched to intravenous pentamidine (from TMP-SMX) and ten patients were switched to clindamycin and primaquine (from TMP-SMX). The drugs were most often switched due to intolerance (e.g. rash, hepatotoxicity) and were less often switched due to clinical failure.

**Figure 1.**
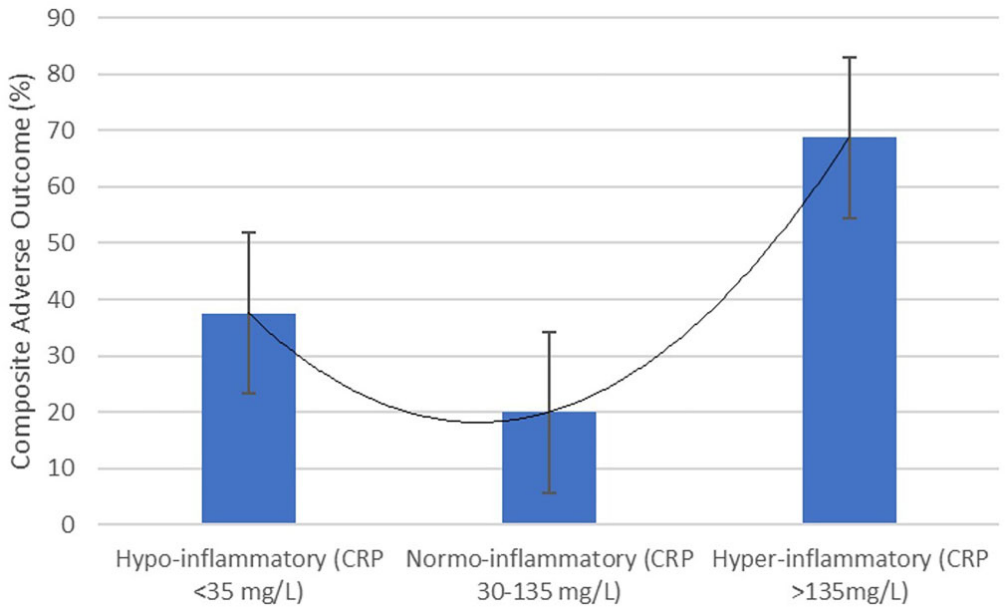
Composite adverse outcomes (death or mechanical ventilations) across the three groups of hypo-, normo-, and hyper-inflammatory patients with *Pneumocystis jirovecii* pneumonia without underlying HIV.

Mortality was higher in the hyperinflammatory group (62.5%), compared with the normo-inflammatory and hypo-inflammatory groups (13.3%, 6.3%, respectively, *P* < 0.001) (Table 1). In terms of composite adverse outcomes, there appeared to be a U-shaped relationship (Fig. 1). The normo-inflammatory group had the best outcomes (20.0%), compared with a higher prevalence of adverse outcomes in the hyper-inflammatory group (62.5%), and hypo-inflammatory group (37.5%, *P* = 0.005). Additionally, on subsequent multivariable analyses, an elevated C-reactive protein>135 mg/l (representing the hyper-inflammatory group) appeared to be independently associated with composite adverse outcomes (adjusted odds ratio 6.98, 95% confidence interval 1.81–26.92, *P* = 0.005), even after adjusting for the effect of older age and male sex (Table 2).

**Table 2.** Multivariable analysis showing predictors of adverse outcome (death or mechanical ventilation) in patients with *Pneumocystis pneumonia* without underlying HIV

Parameter	Adjusted odds ratio (95% confidence interval)	*P*-value
**Older age (>65 years old)**	1.27 (0.40–4.03)	0.684
**Male sex**	0.63 (0.19–2.10)	0.449
**Elevated C-reactive protein (>135 mg/l)**	6.98 (1.81–26.92)	0.005

## Discussion

Inflammatory phenotypes in association with prognosis and outcomes has been described in several infectious diseases. Most recently, it had been described in acute SARS-CoV-2 infection (Ngiam et al. 2023). Latent class analyses of a large cohort of critically ill patients with SARS-CoV-2 infection demonstrated not only unique prognostic outcomes associated with each phenotype, but also identified specific sub-phenotypes with more favourable response to corticosteroid therapy. In such critically ill patients, it may be important to clearly delineate the patients who would benefit most from corticosteroid therapy. Indiscriminate use of corticosteroids in all patients may increase the risk of ventilator associated pneumonia and lead to poorer outcomes overall (Mesland et al. 2022).

We hypothesize that these concepts may also be similarly applied to PJP. Patients with PJP who have underlying acute leukaemia, in addition to further immunosuppressive therapy may be profoundly neutropenic, and unable to mount an immune response to infection. Comparatively, a patient with solid organ cancer on first-line chemotherapy who acquires PJP may be able to produce a large immune response to the infection. These patients are likely to have vastly different outcomes from PJP, and may also have differential response to corticosteroid therapy.

True enough, by arbitrarily dividing patients with PJP by CRP centiles, we showed that both the hypo-inflammatory group with a blunted immune response (as evidenced by a low CRP) and hyper-inflammatory group with an exaggerated immune response (as evidence by a markedly elevated CRP) had poorer outcomes compared with the normo-inflammatory group. The hypo-inflammatory group may represent a group with more profound immunosuppression that resulted in poorer outcomes, while the hyper-inflammatory group may represent a more severe infection, potentially with a larger load of PJP and a consequently more pronounced immune response.

We were not able to demonstrate the differential effect of corticosteroid therapy across the three groups, due to the retrospective nature of our study design and the relatively small numbers in each group. Nevertheless, we believe that our findings are exploratory and hypothesis-generating. We stratified patients with PJP by their inflammatory status, and showed differential outcomes amongst the groups.

## Limitations

There remained several further limitations to our findings. The sample size was small, and reflecting a single centre experience over a long period of time. Improvement in intensive care and other uncontrolled factors may have thus also have contributed to the differences in outcomes. The retrospective nature of our study, and the fact that data had been obtained from review of medical records also has several limitations. Firstly, we used all-cause in-hospital mortality, had not been able to define attributable mortality to PJP. Nevertheless, all patients presented with clinical symptoms and imaging findings consistent with PJP. By design, diagnostic tests were also determined by the primary treating physicians. As such, adjunctive tools for diagnosis like serum beta-D-glucan was rarely sent due to a long turnaround time in our clinical setting. Only 12 patients had serum beta-D-glucan sent, of which 7 tests were positive. Additionally, we had not captured data on the FiO_2_ for each patient, and therefore could not present SaO_2_/FiO_2_ ratios to better characterize disease severity.

Furthermore, the patients studied were highly heterogenous in terms of the types of immunosuppression, and larger studies would be needed to stratify patients by their immunosuppressive regimen to examine the effect of this parameter. We had also deliberately included patients with a presumptive diagnosis of PJP as this reflected real-world experience. A significant proportion of patients with PJP may have had severe coagulopathy or have been too critically ill to undergo bronchoscopy, or may have declined the procedure. This would have precluded a definitive diagnosis of PJP in a significant proportion of patients. Additionally, we only studied one biomarker. To better define inflammatory phenotypes, future prospective study may evaluate novel biomarkers and inflammatory pathways. For example, the STAT3 pathway may be important in the pathophysiology of PJP (Charpentier et al. 2021). Additionally, novel approaches such as latent class analyses using a combination of different biomarkers may also help to more precisely define these phenotypes in association with adverse outcomes (Sinha et al. 2021). Future prospective study is needed to confirm and define the distinct inflammatory phenotypes in PJP, and subsequently examine the role potential role of adjunctive corticosteroids and other targeted therapeutics in relation to these phenotypes in improving patient outcomes.

## Conclusions

This study raises the possibility of a U-shaped relationship between C-reactive protein and clinical outcomes in patients with PJP without underlying HIV. Patients from both ends of the inflammatory spectrum had increased adverse outcomes in our study. These findings invite further study on the influence of degree of immunosuppression on outcomes and the role of adjunctive corticosteroid therapy amongst non-HIV infected immunocompromised hosts with PJP.

## Data Availability

Data may be made available on reasonable request from the corresponding author.
